# Evaluation of Root-End Resections Performed by Er, Cr: YSGG Laser with and without Placement of a Root-End Filling Material

**DOI:** 10.1155/2009/798786

**Published:** 2009-08-06

**Authors:** John Sullivan, Roberta Pileggi, Claudio Varella

**Affiliations:** Department of Endodontics, University of Florida, 1600 SW Archer Rd. Room no. D10-37, Gainesville, FL 32610, USA

## Abstract

Microleakage following root-end resections has a direct influence on the outcome of surgical endodontic procedures. This study compared the microleakage after root-end resections performed by the Er, Cr: YSGG laser or carbide burs with or without the placement of MTA, and evaluated the presence of microcracks and gaps at the interface of GP/MTA and the canal walls. Ninety single-rooted teeth were instrumented, obturated with GP and AH-Plus sealer, and divided into 3 experimental groups: (I) root-end resections were performed with the laser and G6 tips (parameters: 4.5 w, 30 pps, 20% water and 50% air); (II) Lindeman burs were used, without the placement of MTA; (III) the burs were used followed by root-end fillings with MTA, and one control (IV) of five unobturated roots resected with the burs. The samples were prepared for microleakage (*n* = 20) and SEM (*n* = 10) analysis. They were immersed in 1% methylene blue, decalcified, cleared, and evaluated for dye penetration (mm^2^) with the ImageJ software. Epoxy-resin replicas of the root-ends were analyzed by SEM for gaps (*μ*m^2^) and microcracks. Microleakage results were 0.518 ± 1.059, 0.172 ± 0.223, and 0.158 ± 0.253, for the laser (I), no root-end filling (II), and MTA (III) samples, respectively, (ANOVA *P* = .02). The laser (7831.7 ± 2329.2) and no root-end filling (7137.3 ± 1400.7) samples presented gaps. Whereas, none was found in the MTA (ANOVA *P* = .002). Microcracks were not observed. The MTA group demonstrated statistically less leakage and better adaptation to the canal walls when compared to the other groups. There was no correlation between the size of the gaps and the degree of microleakage.

## 1. Introduction

Root-end resection may be the treatment of choice for teeth in which adequate nonsurgical retreatment had failed to eliminate existing periapical pathosis [1, 2]. Thus, following the resection, efforts should be made to seal the root-ends and prevent apical microleakage, improving the outcome of surgical endodontic procedures [3, 4].

The surgical procedures target the elimination of the etiological factor of failure (typically microbial) and the sealing of the root canal system apically, promoting healing of the periradicular tissues [1, 5]. Removal of the last three millimeters of the root eliminate most of the apical deltas, isthmuses, and other canal irregularities, usually present at that specific area of the root canal system. Consequently, the microorganisms harbored in these canals are removed, preventing the seepage of their byproducts to the periapical tissues [6].

Carbide burs mounted on high-speed hand-pieces provide adequate smooth surfaces for root-end resections. However, little is known if the type of bur used or the degree of smoothness after root-end resection would have a significant impact on the clinical outcome of surgical endodontics [7].

Root-end resection performed with laser result in ablation of the exposed dentinal tubules [8], which may decrease microleakage, and increase the resistance to root resorption [9]. The absence of vibration [10], during the root-end resection with lasers may also prevent loss of adaptation between the gutta-percha and the canal wall.

Elimination of microorganism is another important component of the endodontic surgical procedure, and the Er, Cr: YSGG lasers have been shown to be effective against *E. faecalis*, an important pathogen in cases of persistent periapical lesions [11, 12].

Different root-end filling materials have been utilized in surgical procedures after root-end resection and preparation in an attempt to prevent microleakage and promote bone healing [13, 14]. However, their placement may not be feasible in certain clinical situations where excessive bleeding or restricted access and visualization are expected. Accordingly, searching for alternative techniques that may provide adequate root-end resections and apical seal might be of clinical relevance.

The hypothesis of the present study is that root-end resections performed with the Er, Cr: YSGG laser and without the placement of retrofilling material result in leakage similar to that observed when the procedure is performed with surgical carbide burs and the mineral trioxide aggregate (MTA) is used as root-end filling material. Therefore, the aim of this study was twofold. (1) To compare microleakage in root-end resections performed either with Er, Cr: YSGG laser or surgical carbide burs with and without the placement of the root-end filling material. (2) To evaluate the adaptation of the gutta-percha and the MTA to the root canal walls by measuring the gap between these root-end filling materials and the dentinal walls, and the presence of cracks in the resected surfaces.

## 2. Material and Methods

The sample of this study consisted of ninety-five maxillary and mandibular anterior straight single-rooted teeth. The samples were decoronated prior to cleaning and shaping. The cervical and middle thirds of the canals were preflared with Gates-Gliddens drills (Moyco Union Broach, York, PA) numbers 4, 3, and 2, in that sequence. ProFile 0.06 (Dentsply-Maillefer, Tulsa, OK) ISO rotary instruments were lubricated with RC-Prep (Premier Dental Products Co, Plymouth Meeting, PA) and used to prepare the canals to a master apical file size 40. All canals were then obturated with a fine-medium (FM) nonstandardized gutta-percha cone and AH-Plus root canal sealer (Dentsply-Maillefer, Tulsa, OK). Downpack was performed with System B unit (SybronEndo, Orange, CA), and Obtura II (Obtura Corporation, Fenton, MO) was used for incrementally backfilling the middle and cervical thirds of the canal. Schilder pluggers (Dentsply, Tulsa, OK) were used to aid the adaptation of the filling material to the root canal walls.

After obturation, the samples were maintained in 100% humidity for 72 hours. They were then randomly assigned to one of the three experimental groups, containing 30 samples each or the control group (*n* = 5). Group I, the root-end resections were performed with the Er, Cr: YSGG laser (Waterlase MD, Biolase Technology, Inc, San Clement, CA), using hard tissue mode and a G6 tip with the following parameters: 4.5 w, 30 pps, 20% water and 50% air. The laser tip was placed perpendicularly to the long axis of the roots, 3.0 mm from the root apex, in order to obtain root-end resection without a bevel. After resection, the gutta-percha was cold-burnished with a ball-ended plugger (EIE, San Diego, CA). This group did not receive the root-end filling material. Group II, the root-end resections were performed with Lindeman carbide burs (Brasseler, Savanna, GA), also positioned perpendicular to the long axis of the root to prevent beveling. The gutta-percha was cold burnished the same way as described for the teeth in group I and no root-end filling was used in this group. Group III, the root-end resections were performed with the Lindeman burs (0° bevel), followed by root-end preparation with the MiniEndo ultrasonic unit (SybronEndo Corp., Orange, CA) and a KiS 1D tip (Obtura/Spartan, Fenton, MO). Mineral trioxide aggregate (ProRoot MTA Dentsply/Tulsa Dental, Tulsa, OK) was used as the root-end filling material. The MTA was placed into the prepared root-end cavities with the aid of the MAP system (Produits Dentaires, Vevey, Switzerland) and condensed with micropluggers (EIE, San Diego, CA). Group IV (positive control), five instrumented but unobturated roots were resected with Linderman burs, root-end preparation was performed and no root-end filling material was placed. This should allow for maximum dye penetration into the canals. The teeth from all groups were left in 100% humidity for 72 hours and prepared for either leakage or SEM analysis. Out of the 30 samples from each group, 20 were prepared for clearing and microleakage analysis and 10 were used for SEM gap measurements.

### 2.1. Microleakage Samples

**Figure 1:**
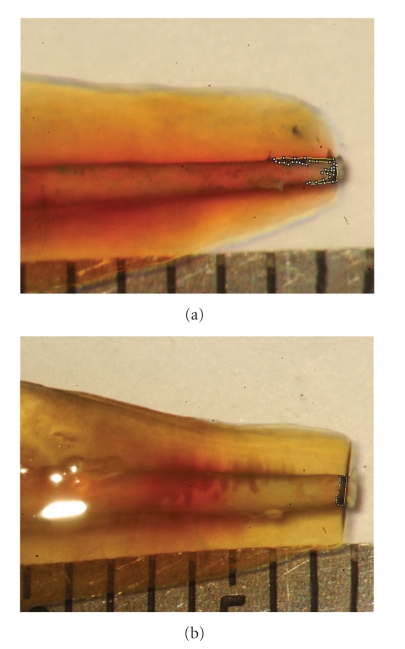
Digital photographs of cleared samples demonstrating the measurements of leakage penetration with the aid of the ImageJ software.

The samples were coated with nail polish (Procter & Gumble Distr., Hunt Valley, MD) leaving exposed only the resected surfaces and placed in suspension overnight (18 hours) in 1% methylene blue dye (LabChem Inc., Pittsburg, PA) at room temperature. Before clearing procedures, the roots were carefully cleaned and the nail polish was removed with the aid of periodontal curettes and nail polish remover. They were decalcified at room temperature for two days in decalcifying solution (Richard-Allan Scientific, Kalamazoo, MI), rinsed with tap water and dehydrated in ethyl alcohol (Fisher Chemicals, Fair Lawn, NJ). The samples were left overnight in an 80% solution, followed by one-hour submersion in 90%, and finally one hour in 100% ethyl alcohol. The samples were then rendered transparent by soaking in methyl salicylate (Fisher Chemicals, Fair Lawn, NJ) for two days. Digital photographs were obtained from the mesial and distal aspects of each sample. The images' sizes were increased by 200% to facilitate data analysis (Figures 1(a) and 1(b)). Two evaluators, unaware from which group the specimens came from, measured the areas of dye penetration with the aid of ImageJ software (National Institute of Health, Bethesda, MD).

### 2.2. Gap Measurement (SEM) Samples

**Figure 2:**
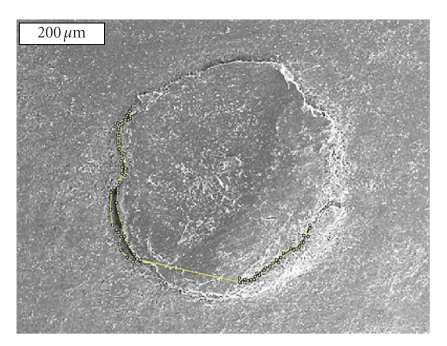
Scanning Electron Microscope picture of the resected root-end (x60) showing the gap measurement between the gutta-percha and the root canal wall using the ImageJ software.

The scanning electron microscope (SEM) samples were prepared by creating epoxy resin replicas from polyvinylsiloxane impressions of the resected root-ends. The resin replica technique was used in this study to eliminate artifacts linked to SEM processing [15]. The ability of the resin replica to duplicate detail was tested by placing microscratches in the resected root-end of a natural tooth and then fabricating a resin replica. The resin replica and test specimen were placed on an SEM mounting stub next to one another, and the amount of detail in corresponding areas was compared under high-power magnification. All replicas were mounted on aluminum stubs adjacent to the natural specimen. This was done to ensure the accuracy of the resin replica process was consistently able to reproduce surface details present in corresponding areas of the natural tooth and if any detail was lost or any resin processing artifacts were present, then that natural tooth was used for comparison purposes. After allowing them to set for 24 hours, the resin replicas were kept overnight in a disecator under vacuum, then coated with platinum, and analyzed under the SEM (JOEL JSM 6400, JOEL Ltd. Peabody, MA) at x60 magnification. Photomicrographs were obtained for the evaluation of the gutta-percha/MTA adaptation at the root canal interface using the ImageJ software (Figure 2). The presence of microcrack formation was also recorded.

The leakage and gap measurements from the two evaluators were averaged and analyzed statistically using ANOVA and Scheffe post hoc test for any significant differences between the groups. The significance level was set at *P* ≤ .05.

## 3. Results

**Figure 3:**
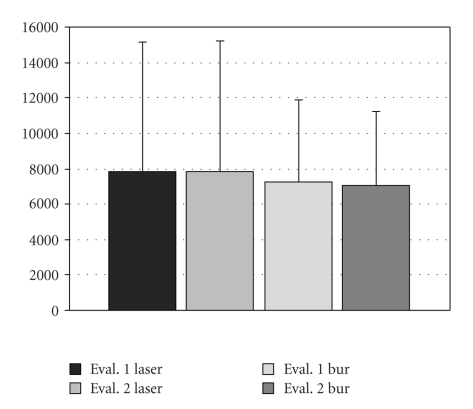
Comparative analysis of average and standard deviation for the Gap measurement between evaluators. The MTA group is not shown because there were no measurable gaps.

**Figure 4:**
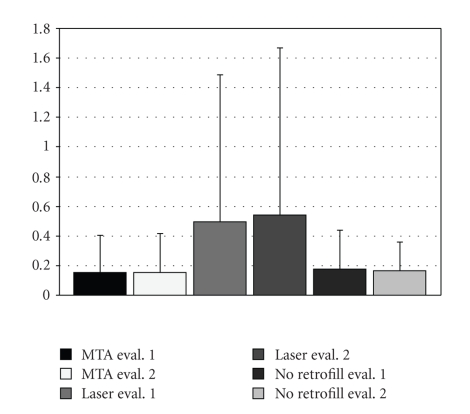
Comparative analysis of average and standard deviations for the leakage measurements between evaluators.

**Figure 5:**
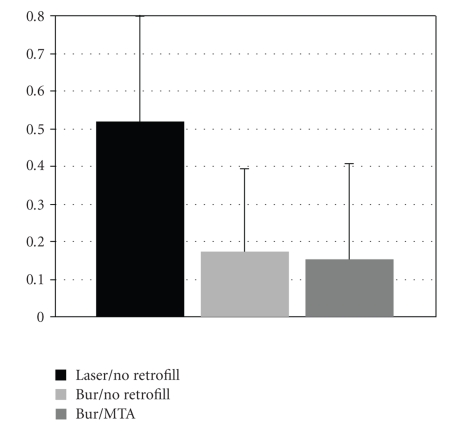
Mean leakage and standard deviation for leakage (mm^2^) for the different groups.

**Figure 6:**
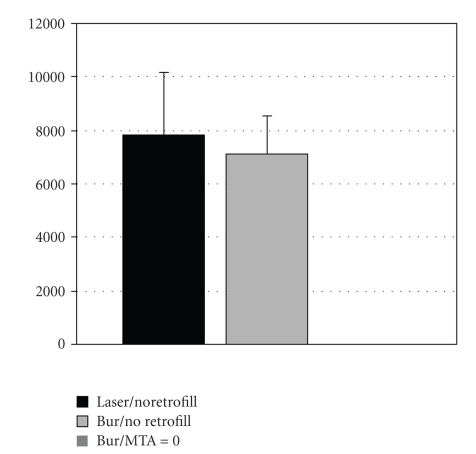
Mean and standard deviation from gap measurements (*μ*m^2^) for the experimental groups.

Interevaluators comparisons are demonstrated in Figures 3 and 4. The mean leakage and gap measurements for each group are shown in Figures 5 and 6, respectively. The leakage data is presented in millimeter square (mm^2^); whereas, the gap measurements are presented in micrometer square (*μ*m^2^). The average and standards deviation for leakage were 0.518 ± 1.059, 0.172 ± 0.223, and 0.158 ± 0.253, for the laser (group I), no root-end filling (group II), and MTA (group III) samples, respectively, (ANOVA *P* = .02). The controls demonstrated dye penetration through the entire length of the root canal. SEM analysis demonstrated measurable gaps in the laser (7831.7 ± 2329.2) and bur resection without root-end filling (7137.3 ± 1400.7) groups. A summary of the results is presented in the Table 1. The MTA group (group III) demonstrated statistically less microleakage when compared with the laser group (group I) and the best marginal adaptation, with no measurable gaps at the MTA-dentinal wall interface, when compared to the other two groups. There was no significant difference between the laser (group I) and the bur root-end resections (group II) for both the microleakage and gap measurements, when the root-end filling material (MTA) was not used.

Microcracks were not observed in any of the specimens analyzed under the SEM.

## 4. Discussion

The study results do not support the hypothesis. Root-end resection performed with the Er, Cr: YSGG laser without the placement of a root-end filling material demonstrated greater amount of leakage than when the procedure was performed with carbide bur and MTA was used as root-end filling material. Thus, gap formation was frequently observed, between the gutta-percha and the root canal walls, in the roots treated with the laser device.

Comparison of the root-end resected surface topography when the bur or the laser was used also demonstrated remarkable differences. The laser device provided a much rougher inconsistent surface. This may be a problem because it increases the resected root-end surface area and theoretically will expose more dentinal tubules than a smooth flat resected surface. It is possible that the laser does obliterate tubules [8]; however, the increase in surface area counteracts the obliteration. Thus, it may also make more difficult to clinically inspecting the resected root-end for vertical root fractures and burnish the retrofill material smooth at the margins.

The placement of a root-end filling material may not play an essential role in the outcome of surgical endodontics if the etiological factor (i.e., microorganism at the apical third) is eliminated by the root-end resection [16]. Nonetheless, some investigators [17] had supported that the prognosis of surgical cases improve whenever a root-end filling material is used.

The laser setting may also have an effect on the ability of the laser to obliterate tubules. Different power levels and pulse rates will either obliterate or open dentinal tubules [18, 19]. The settings used were based on the pilot data from another study (unpublished data) by our group. However, further investigation is needed to evaluate the dentin effect of the different Er, Cr: YSGG laser settings.

Currently, the mineral trioxide aggregate (MTA) seems to be the material of choice for root-end fillings due to its mechanical and biological properties [13, 20, 21]. MTA has shown better sealing ability [22, 23] and biological responses when compared to other commonly used root-end filling materials, such as Super-EBA, amalgam, and IRM [20, 24]. The group that received MTA as the root-end filling material demonstrated the least amount of microleakage, which is in agreement with previous reports [25, 26]. However, the samples were allowed to set in 100% humidity for 72 hours prior to the leakage study, and it may not completely represent the clinical scenario. The MTA has long setting time and could be partially or totally washed out from the root-end cavity before it is completely set, impairing it's sealing ability. In contrastthough, it should be noted that the sealing ability of MTA increases as it stimulates the formation of hydroxyapatite filling in the microscopic voids between the MTA and dentinal wall [27].

The resin replica proved to be representative of the natural tooth by consistently reproducing the details. They proved to be beneficial in differentiating lack of marginal adaptation versus artifactual cracking. Thus, the cracks observed in the natural teeth were most likely caused from the vacuum and dehydration while being prepared for SEM analysis. None of the replicas demonstrated the presence of cracks.

The present study also showed no correlation between the presence of gaps and the degree of microleakage, which is in agreement with previously published reports [28–30]. Even though dye penetration studies have been widely used to evaluate the sealing ability of endodontic filling materials [31], they have limitations and their results should be analyzed carefully and not directly extrapolated to the clinical situation. Dye penetration is not uniform around the margins of the root-end filling material, which may render unreliable measurements [32]. In the present study, to eliminate this problem, rather than providing linear measurements, we measured the total area of dye penetration from both mesial and distal aspects of the root.

The other problem with dye studies may be related to the molecular size of the dye or the bacteria used in the study design and to the fact that dye solutions cannot duplicate the bacterial behavior within the root canal system [31].

In summary, based on the parameter of this in vitro study, we can conclude that root-end resections performed with bur and MTA used as the root-end filling material demonstrated statistically significant less microleakage and better canal wall adaptation when compared to root-end resection performed with bur or laser without a root-end filling.

## Figures and Tables

**Table 1:** Comparison of mean dye leakage and gap presence in the experimental root-end resected groups.

Technique	Leakage ± SD (*n* = 20)	Gap ± SD (*n* = 10)
	(mm^2^)	(*μ*m^2^)
Laser (cold burnish)	0.518 ± 1.059	7831.7 ± 2329.2
Carbide bur (cold burnish)	0.172 ± 0.223	7137.3 ± 1400.7
Carbide bur (MTA root-end filling)	0.158 ± 0.253	0

## References

[1] Lin S, Platner O, Metzger Z, Tsesis I (2008). Residual bacteria in root apices removed by a diagonal root-end resection: a histopathological evaluation. *International Endodontic Journal*.

[2] Abramovitz I, Better H, Shacham A, Shlomi B, Metzger Z (2002). Case selection for apical surgery: a retrospective evaluation of associated factors and rational. *Journal of Endodontics*.

[3] Torabinejad M, Hong C-U, Lee S-J, Monsef M, Pitt Ford TR (1995). Investigation of mineral trioxide aggregate for root-end filling in dogs. *Journal of Endodontics*.

[4] Montellano AM, Schwartz SA, Beeson TJ (2006). Contamination of tooth-colored mineral trioxide aggregate used as a root-end filling material: a bacterial leakage study. *Journal of Endodontics*.

[5] Nair PNR, Sjögren U, Figdor D, Sundqvist G (1999). Persistent periapical radiolucencies of root-filled human teeth, failed endodontic treatments, and periapical scars. *Oral Surgery, Oral Medicine, Oral Pathology, Oral Radiology, and Endodontics*.

[6] Kim S, Kratchman S (2006). Modern endodontic surgery concepts and practice: a review. *Journal of Endodontics*.

[7] Morgan LA, Marshall JG (1998). The topography of root ends resected with fissure burs and refined with two types of finishing burs. *Oral Surgery, Oral Medicine, Oral Pathology, Oral Radiology, and Endodontics*.

[8] Kimura Y, Wilder-Smith P, Matsumoto K (2000). Lasers in endodontics: a review. *International Endodontic Journal*.

[9] Paghdiwala AF, Vaidyanathan TK, Paghdiwala MF (1993). Evaluation of erbium:YAG laser radiation of hard dental tissues: analysis of temperature changes, depth of cuts and structural effects. *Scanning Microscopy*.

[10] Komori T, Yokoyama K, Takato T, Matsumoto K (1997). Clinical application of the erbium: YAG laser for apicoectomy. *Journal of Endodontics*.

[11] Schoop U, Kluger W, Moritz A, Nedjelik N, Georgopoulos A, Sperr W (2004). Bactericidal effect of different laser systems in the deep layers of dentin. *Lasers in Surgery and Medicine*.

[12] Wang Q, Zhang C, Yin X (2007). Evaluation of the bactericidal effect of Er,Cr:YSGG, and Nd:YAG lasers in experimentally infected root canals. *Journal of Endodontics*.

[13] Torabinejad M, Chivian N (1999). Clinical applications of mineral trioxide aggregate. *Journal of Endodontics*.

[14] Niederman R, Theodosopoulou JN (2003). A systematic review of in vivo retrograde obturation materials. *International Endodontic Journal*.

[15] Crang RFE, Klomparens KL (1988). Artifacts in biological electron microscopy. *Science*.

[16] Johnson BR (1999). Considerations in the selection of a root-end filling material. *Oral Surgery, Oral Medicine, Oral Pathology, Oral Radiology, and Endodontics*.

[17] Altonen M, Mattila K (1976). Follow up study of apicoectomized molars. *International Journal of Oral Surgery*.

[18] Corona SAM, de Souza AE, Chinelatti MA, Borsatto MC, Pécora JD, Palma-Dibb RG (2007). Effect of energy and pulse repetition rate of Er: YAG laser on dentin ablation ability and morphological analysis of the laser-irradiated substrate. *Photomedicine and Laser Surgery*.

[19] Zapletalová Z, Perina J, Novotný R, Chmelícková H (2007). Suitable conditions for sealing of open dentinal tubules using a pulsed Nd:YAG laser. *Photomedicine and Laser Surgery*.

[20] Bernabé PFE, Holland R, Morandi R (2005). Comparative study of MTA and other materials in retrofilling of pulpless dogs’ teeth. *Brazilian Dental Journal*.

[21] Yoshimine Y, Ono M, Akamine A (2007). In vitro comparison of the biocompatibility of mineral trioxide aggregate, 4META/MMA-TBB resin, and intermediate restorative material as root-end-filling materials. *Journal of Endodontics*.

[22] Pelliccioni GA, Vellani CP, Gatto MRA, Gandolfi MG, Marchetti C, Prati C (2007). Proroot mineral trioxide aggregate cement used as a retrograde filling without addition of water: an in vitro evaluation of its microleakage. *Journal of Endodontics*.

[23] Storm B, Eichmiller FC, Tordik PA, Goodell GG (2008). Setting expansion of gray and white mineral trioxide aggregate and Portland cement. *Journal of Endodontics*.

[24] Sarris S, Tahmassebi JF, Duggal MS, Cross IA (2008). A clinical evaluation of mineral trioxide aggregate for root-end closure of non-vital immature permanent incisors in children-a pilot study. *Dental Traumatology*.

[25] Fischer EJ, Arens DE, Miller CH (1998). Bacterial leakage of mineral trioxide aggregate as compared with zinc-free amalgam, intermediate restorative material, and Super-EBA as a root-end filling material. *Journal of Endodontics*.

[26] Mangin C, Yesilsoy C, Nissan R, Stevens R (2003). The comparative sealing ability of hydroxyapatite cement, mineral trioxide aggregate, and super ethoxybenzoic acid as root-end filling materials. *Journal of Endodontics*.

[27] Sarkar NK, Caicedo R, Ritwik P, Moiseyeva R, Kawashima I (2005). Physicochemical basis of the biologic properties of mineral trioxide aggregate. *Journal of Endodontics*.

[28] Abdal AK, Retie DH, Jamison HC (1982). The apical seal via the retrosurgical approach. II. An evaluation of retrofilling materials. *Oral Surgery, Oral Medicine, Oral Pathology*.

[29] Yoshimura M, Marshall FJ, Tinkle JS (1990). In vitro quantification of the apical sealing ability of retrograde amalgam fillings. *Journal of Endodontics*.

[30] Xavier CB, Weismann R, de Oliveira MG, Demarco FF, Pozza DH (2005). Root-end filling materials: apical microleakage and marginal adaptation. *Journal of Endodontics*.

[31] Barthel CR, Moshonov J, Shuping G, Orstavik D (1999). Bacterial leakage versus dye leakage in obturated root canals. *International Endodontic Journal*.

[32] Camps J, Pashley D (2003). Reliability of the dye penetration studies. *Journal of Endodontics*.

